# Building bridges between body and mind: liaison psychiatry

**DOI:** 10.1192/j.eurpsy.2022.1200

**Published:** 2022-09-01

**Authors:** S. Bravo Herrero, I. Moreno Alonso, M.J. Sánchez Artero, A.M. Matas Ochoa

**Affiliations:** 1 Hospital Universitario Infanta Leonor, Liaison Psychiatry, Madrid, Spain; 2 Hospital Universitario Infanta Leonor, Home Treatment Team, Madrid, Spain

**Keywords:** liaison, coordination, psychiatry, physicians

## Abstract

**Introduction:**

As liaison psychiatrists, it is very important to mantein a good relationship with other medical specialties in order to obtain the best result for our patients. Most of the times, the somatic process affects direct or indirectly to mental healt and vice versa, so our cooperation is extremely important for the patient’s welbeing.

**Objectives:**

With this study we try to find special considerations and necesities of every specialty that count on us in our hospital. We have design this batebase with the aim of discovering which are the main problems that suffer the admitted patients, which doubts face our colleagues when evaluate mental health patients, etc. Thus, our team could help other physicians properly or so we could stablish a proper liaison in order to make things easier.

**Methods:**

A database has been created with all the patients evaluated by our liaison psychiatry team during half a year. We have taken into account sex, age, referral specialist, mental health diagnosis (after our evaluation), previous mental health follow-up, if they are on psycopharmacology treatment, if they requiere psycopharmacology treatment and if they requiere follow-up once discharged.

**Results:**

22,9% were kid/adolescent patients. 25,8% were elderly people (>70 yo). 47% were men (of which, 6% were trans men), 53% were women. 22,9% suffered from adjustment disorder, 14,1% had no acute mental health problem, 11,76% presented substance abuse. Main petitions were made from Internal Medicine (30%)

**Conclusions:**

With this information we can explore other specialists’ and admitted patients’ needs and concerns and focus our effort in solving them.

**Disclosure:**

No significant relationships.

